# Incidence and general hospital costs of self-harm across England: estimates based on the multicentre study of self-harm

**DOI:** 10.1017/S2045796020000189

**Published:** 2020-03-12

**Authors:** Apostolos Tsiachristas, Galit Geulayov, Deborah Casey, Jennifer Ness, Keith Waters, Caroline Clements, Nav Kapur, David McDaid, Fiona Brand, Keith Hawton

**Affiliations:** 1 Health Economics Research Centre, Nuffield Department of Population Health, University of Oxford, Oxford, UK; 2 Centre for Suicide Research, Department of Psychiatry, Warneford Hospital, University of Oxford, Oxford, UK; 3 Centre for Self-harm and Suicide Prevention Research, Derbyshire Healthcare NHS Foundation Trust, Derby, UK; 4 Centre for Suicide Prevention, Manchester Academic Health Sciences Centre, University of Manchester, Manchester, UK; 5 Greater Manchester Mental Health NHS Foundation Trust, Manchester, UK; 6 Department of Health Policy, Personal Social Services Research Unit, The London School of Economics and Political Science, London, UK; 7 Oxford Health NHS Foundation Trust, Oxford, UK

**Keywords:** Economic issues, emergency departments, health economics, incidence, suicide

## Abstract

**Aims:**

The aim of this study was to estimate incidence of self-harm presentations to hospitals and their associated hospital costs across England.

**Methods:**

We used individual patient data from the Multicentre Study of Self-harm in England of all self-harm presentations to the emergency departments of five general hospitals in Oxford, Manchester and Derby in 2013. We also obtained cost data for each self-harm presentation from the hospitals in Oxford and Derby, as well as population and geographical estimates from the Office for National Statistics. First, we estimated the rate of self-harm presentations by age and gender in the Multicentre Study and multiplied this with the respective populations to estimate the number of self-harm presentations by age and gender for each local Clinical Commissioning Group (CCG) area in England. Second, we performed a regression analysis on the cost data from Oxford and Derby to predict the hospital costs of self-harm in Manchester by age, gender, receipt of psychosocial assessment, hospital admission and type of self-harm. Third, the mean hospital cost per age year and gender were combined with the respective number of self-harm presentations to estimate the total hospital costs for each CCG in England. Sensitivity analysis was performed to address uncertainty in the results due to the extrapolation of self-harm incidence and cost from the Multicentre Study to England.

**Results:**

There were 228 075 estimated self-harm presentations (61% were female) by 159 857 patients in 2013 in England. The largest proportions of self-harm presentations were in the age group 40–49 years (30%) for men and 19–29 years (28%) for women. Associated hospital costs were approximately £128.6 (95% CI 117.8−140.9) million in 2013. The estimated incidence of self-harm and associated hospital costs were lower in the majority of English coastal areas compared to inland regions but the highest costs were in Greater London. Costs were also higher in more socio-economically deprived areas of the country compared with areas that are more affluent. The sensitivity analyses provided similar results.

**Conclusions:**

The results of this study highlight the extent, hospital costs and distribution of self-harm presentations to hospitals in England and identify potential sub-populations that might benefit from targeted actions to help prevent self-harm and assist those who have self-harmed. They can support national as well as local health stakeholders in allocating funds and prioritising interventions in areas with the greatest need for preventing and managing self-harm.

## Introduction

Self-harm, increasingly acknowledged as a major public health concern (Borschmann *et al*., 2018; Pilling *et al*., 2018; The Lancet Public, 2018; Ayre *et al*., 2019), is a key area in the national suicide prevention strategies of many countries and is a priority area in the Mental Health Gap Action Programme produced by the World Health Organization (World Health Organization, 2008). People who self-harm are at elevated risk of premature death (Hawton *et al*., 2006; Bergen *et al*., 2012; Carr *et al*., 2017), especially by suicide (i.e. death by intentional self-harm) (Bergen *et al*., 2012; Carroll *et al*., 2014; Olfson *et al*., 2018), and poor mental health, including depression and substance abuse (Da Cruz *et al*., 2011; Mars *et al*., 2014; Borschmann *et al*., 2017).

In England, prevention of self-harm and suicide is a priority area in public health policy, being the focus of national strategy and clinical guidelines (NICE, 2011; UK Government, 2012, 2019). It was highlighted as a key issue in its own right when the national suicide prevention strategy in England was updated in 2017 and its prevention was recognised as fundamental priority for all organisations involved in delivering the strategy (HM Government, 2019). Furthermore, the first ever Minister of Mental Health, Inequalities and Suicide Prevention was appointed in 2018 along with increased funding for suicide prevention (GOV.UK, 2018). In a series of policy initiatives, local NHS organisations and local government have been asked to draw up joint plans, according to guidelines from Public Health England, to reduce suicide by 10% in 2020 (Appleby *et al*., 2017; NHS England, 2018). Although suicide rates are strongly related to self-harm rates (Geulayov *et al*., 2018), hospital management of self-harm remains variable across the country and there has until recently been little sign of service improvement over time (Cooper *et al*., 2013).

Although the overall incidence of self-harm in England has been estimated previously (Hawton *et al*., 2007; Geulayov *et al*., 2016), little is known about its distribution across England. The only available nationwide estimates of self-harm incidence at local level are reported by Public Health England based on hospital admissions, which underestimate the scale of the problem (Clements *et al*., 2016; Public Health England, date accessed 27/02/2018). Besides the impact on population health, self-harm has considerable implications for healthcare costs, including costs of medical, psychiatric and social care (Sinclair *et al*., 2011*a*). A recent UK study based on a single centre estimated hospital costs to be on average £809 per self-harm presentation, with an approximate extrapolation to England of an impact on the NHS budget of approximately £162 million each year (Tsiachristas *et al*., 2017). This is a concerning figure for local health service commissioners, which increasingly face budget constraints and pressure to improve efficiency in healthcare organisation and delivery.

Estimating the incidence of self-harm presentations to hospitals and the associated hospital costs at a local level is key for designing services for individuals who self-harm and in planning hospital budgets. The aim of this study was to estimate the incidence of self-harm presentations to hospitals at both local and national levels and the associated hospital costs across England.

## Methods

### Study setting and primary data

The data were collected as part of the Multicentre Study of Self-harm in England. The three centres in the study have been collecting comprehensive data on hospital presentations for self-harm for many years, using similar methodology. The Multicentre Study of Self-harm in England was established early this century in order to provide more representative data on self-harm than each individual centre could provide. In this respect the three cities have a broad geographical distribution, with Oxford in South-East England, Derby in the East-Midlands and Manchester in North-West England. Oxford, Manchester and Derby also have distinctly different profiles in terms of the extent of socio-economic deprivation of their individual catchment areas. Based on the 2015 ratings of the Index of Multiple Deprivation scores for England, which range from 1 (worst) to 209 (best) across England, Manchester was ranked 5 (worst), Derby 55 and Oxford 166 (Department for Communities and Local Government, 2015). While this does not entirely ensure that the study is fully representative of England as a whole, it means that the data on self-harm are far more representative than those from single centres.

The provision of mental health care in general hospitals in England is mainly limited to that focussed on general medical patients with mental health problems and patients who present following self-harm. This includes both care while patients are in hospital and coordinating care after hospital discharge, such as psychological support (e.g. for cancer patients). The overall provision of mental health-related care is funded through general government funds allocated to NHS England. With regards to self-harm, the National Institute for Health and Care Excellence (NICE) recommends provision of a psycho-social assessment for all patients who present with self-harm to the emergency departments of general hospitals (NICE, 2011). This assessment is conducted by a member of the hospital mental health team and is focussed on assessing patients' problems, needs and risks to determine their subsequent care after leaving hospital. As other specialised mental health care is generally provided by separate community and other mental health teams and is therefore not part of our study. Since there are virtually no emergency departments in private hospitals in England, the cost of self-harm in private hospitals was not included in our study.

Adopting the working definition of the Multicentre Study of Self-harm in England, which is used nationally in England (NICE, 2011), self-harm was defined as intentional self-injury or self-poisoning, irrespective of type of motivation or degree of suicidal intent. Self-poisoning was defined as the intentional self-administration of more than the prescribed or recommended dose of any drug (e.g. analgesics, antidepressants), and includes poisoning with non-ingestible substances (e.g. household bleach), overdoses of ‘recreational drugs’ and severe alcohol intoxication where clinical staff consider such cases to be acts of self-harm. Self-injury was defined as any injury that has been deliberately self-inflicted (e.g. self-cutting, jumping from height). Identification of cases was determined by clinical and research staff using these criteria.

The data included individual patient level data for all self-harm presentations to the emergency departments of five general hospitals (one in Oxford, three in Manchester and one in Derby) between 1 April 2013 and 31 March 2014. The information collected included: overall self-harm method (i.e. self-poisoning, self-injury, both), specific self-harm method (e.g. cutting, poisoning by specific drugs), hospital admission and patient socio-demographic characteristics (i.e. age, gender and ethnicity). It also included the provision of psychosocial assessment. We also obtained the actual hospital cost (i.e. direct and indirect costs of all hospital services) of each self-harm presentation in our dataset (i.e. in 2013/14 fiscal year) from the finance departments of the hospitals in Oxford and Derby. Mid-year 2013 population estimates for the study catchment areas by single year of age and gender, as well as suicide rates and proportion of the catchment area populations living in rural areas were retrieved at Clinical Commissioning Group level from the Office for National Statistics (ONS). Data on the Market Forces Factor (an index that adjusts price differences across the country) in Oxford, Manchester and Derby were retrieved from NHS England.

### Approximating the incidence of self-harm presentations to hospitals across England

The number of self-harm presentations was divided by the total population in the catchment area of the three centres of the Multicentre Study for single age years and gender to estimate the rate of self-harm presentation to hospital by age and gender in 2013. This rate was multiplied by the population per age year and gender in each local health service commissioning area (known as Clinical Commissioning Groups – CCGs) in England to estimate the number of self-harm presentations in each CCG nationally by age and gender. The total number of self-harm presentations per CCG area in England was calculated by summing all self-harm presentations by age and gender.

### Exploring heterogeneity in hospital costs in the multicentre study

Heterogeneity in costs among hospitals may be explained by patient case-mix (i.e. hospitals provide medical services to patients of different severity and medical needs), mix and quality of services provided (i.e. hospitals may provide services differently for the same need for care and their quality may vary) and production constraints (i.e. hospitals may have different prices for capital and labour inputs) (Street *et al*., 2010). We explored differences in patient case-mix between the three centres in terms of patient socio-demographic characteristics, overall and specific methods of self-harm and number of self-harm presentations during the study period. For this purpose, descriptive statistical analysis (i.e. frequencies, measures of central tendency and variability) was performed and differences between the three centres were tested with ANOVA and Kruskal−Wallis for continuous variables and chi-squares for categorical variables. In a subgroup descriptive analysis, we additionally compared the occupational status of those patients who had received psychosocial assessment between the three centres. Furthermore, we explored the variation in provided services (i.e. hospital admission and provision of psychosocial assessment) across the three centres using a descriptive statistical analysis. Mixed-Effects Generalised Linear Models were specified to estimate odds ratios for hospital admission and provision of psychosocial assessment adjusted for patient case-mix in order to explore differences in quality of care for self-harm between the three centres. Production constraints were accounted in our study by using the Market Forces Factor to adjust for unavoidable and location-specific cost differences (e.g. differences in land, buildings and staff costs) between the hospitals included in the Multicentre Study.

### Estimating hospital costs of self-harm across England

Hospital cost data from Derby did not include the costs of psychosocial assessment. Therefore, we added £392 for patients younger than 18 years and £228 for adult patients to the hospital costs of those patients who had received psychosocial assessment in Derby. These unit costs were published recently and were close to the national average costs of psychosocial assessment reported by the National Institute for Health and Care Excellence (NICE) (Tsiachristas *et al*., 2017). Furthermore, hospital cost data for each self-harm presentation in Oxford and Derby were regressed by gender, age, receipt of psychosocial assessment, hospital admission and general type of self-harm using a generalised linear model with Gamma distribution, log link and standard errors adjusted for clustering of episodes in patients. The coefficients of this regression analysis were fitted to the data from Manchester to estimate the hospital costs of self-harm presentations in Manchester after adjusting further for the Market Forces Factors. Using the hospital cost of all self-harm presentations in the dataset, we then calculated the mean hospital costs per self-harm presentation by age year and gender. The total costs of self-harm in each CCG area in England were then estimated by multiplying the estimated mean hospital costs per self-harm episode by age and gender with the estimated number of self-harm episodes in each CCG by gender and age.

### Sensitivity analysis

Monte-Carlo simulation with 10 000 iterations was performed using the regression coefficients and standard errors from the generalised linear model to address the uncertainty in the results caused by predicting the hospital costs of self-harm presentations in Manchester. The uncertainty based on the simulation was displayed as 95% confidence intervals of the estimated hospitals costs across England. Furthermore, two univariate sensitivity analyses were performed to address the uncertainty in the national estimates of self-harm incidence and related hospital costs from the extrapolation of the Multicentre study. In the first, we used gender-specific and age standardised rates of suicide in each CCG between 2012 and 2014 to adjust the estimated number of self-harm presentations. To do this, we multiplied the estimated number of self-harm presentations by an adjustment factor. The suicide adjustment factor (by gender) was calculated by dividing the age standardised suicide rate in each CCG area by the average age standardised suicide rate in the three centres of the Multicentre Study. The underlying assumption for performing this sensitivity analysis was that suicide (i.e. death by intentional self-harm) and self-harm have common risk factors (Hawton *et al*., 2012) and there is evidence showing a strong positive relationship between rates of self-harm and suicide (Geulayov *et al*., 2018). Given that the method used to estimate the incidence of self-harm in the present study was based on data from largely urban areas in the Multicentre Study, a second univariate sensitivity analysis was performed by adjusting the estimated number of self-harm presentations in each CCG based on the rural/urban classification. For this, we used a rurality adjustment factor (by gender) for each CCG to account for approximately 31% lower self-harm presentations in males and 26% in females in rural areas compared with urban areas in England (Harriss and Hawton, 2011).

### Role of the funding source

The funder of the study reviewed the study proposal, awarded funding and monitored the conduct of the study. The funders had no role in study design, data collection, data analysis, data interpretation or writing of the manuscript. The corresponding author had full access to all the data in the study and had final responsibility for the decision to submit for publication.

## Results

The results in panel A of Table 1 show that the sample in Manchester included proportionally fewer patients younger than 20 years (2 percentage points) and less females (5 percentage points) compared to the other two settings, while there were proportionally more patients of White ethnicity in Oxford (10 percentage points) compared to Manchester and Derby. The percentage of people having two or more self-harm repetitions in 2013 was higher in Derby (9%) followed by Oxford (7%) and Manchester (6%). Among the three centres, the proportion of episodes of self-harm involving self-poisoning alone ranged from 63% in Manchester to 76% in Derby, the proportion in which cutting was the method of self-injury ranged from 65% in Oxford to 80% in Derby, the proportion of self-poisoning episodes involving paracetamol or paracetamol-containing compounds ranged from 27% in Manchester to 33% in Derby (panel B of Table 1). The proportion of self-harm episodes in which a psychosocial assessment was conducted ranged from 50% in Manchester to 73% in Oxford, while admissions to hospitals ranged from 37% of episodes in Manchester to 78% in Oxford. The rate of self-harm presentations per 1000 population was highest in Manchester, except for the age groups 19–29 years, 30–39 years and 60–69 years where it was highest in Derby (panel C of Table 1). More detailed information about the variation in patient case-mix, service provision, self-harm rates and Market Force Factors between the three centres is provided in Appendices 1–5.

**Table 1.** Variation in patients and self-harm episodes across the three centres of the multicentre study

Variable	Oxford	Manchester	Derby
Panel A: Patient characteristics at first self-harm episode			
	*n* (% of 1150)	*n* (% of 3018)	*n* (% of 1548)
Age (years)***			
<18	171 (15)	381 (13)	216 (14)
18–19	80 (7)	196 (7)	116 (8)
20–29	335 (29)	946 (31)	416 (27)
30–39	190 (17)	615 (20)	273 (18)
40–49	188 (16)	499 (17)	312 (20)
50–59	111 (10)	265 (9)	131 (9)
60–69	47 (4)	65 (2)	54 (3)
70 and older	27 (2)	45 (2)	30 (2)
Missing	1 (0)	6 (0)	0 (0)
Sex ***			
Male	446 (39)	1326 (44)	606 (39)
Female	704 (61)	1692 (56)	942 (61)
Ethnicity***			
White	1006 (87)	2313 (77)	1196 (77)
Black	19 (2)	69 (2)	14 (1)
Asian	33 (3)	134 (4)	30 (2)
Other	52 (5)	216 (7)	49 (3)
Missing	40 (3)	286 (10)	259 (17)
Number of self-harm repetitions*			
0	944 (82)	2500 (83)	1240 (80)
1	123 (11)	327 (11)	175 (11)
2	33 (3)	91 (3)	73 (5)
>2	50 (4)	100 (3)	60 (4)
Panel B: Type of self-harm and services provided at all self-harm episodes			
Type of self-harm***	*n* = 1664	*n* = 4078	*n* = 2208
Self-poisoning alone	1155 (69)	2573 (63)	1673 (76)
Self-injury alone	395 (24)	1266 (31)	433 (20)
Both self-poisoning & self-injury	114 (7)	239 (6)	102 (4)
Self-injury method***	*n* = 508	*n* = 1505	*n* = 524
Cutting/stabbing	332 (65)	1024 (68)	422 (80)
Jump from height	11 (2)	33 (2)	8 (2)
Hanging/asphyxiation	45 (9)	162 (11)	45 (9)
Traffic related	5 (1)	47 (3)	3 (1)
Other method#	115 (23)	239 (16)	46 (9)
Self-poisoning	*n* = 963	*n* = 2029	*n* = 226
Paracetamol	214 (22)	431 (21)	376 (27)
Paracetamol compound	57 (6)	124 (6)	76 (6)
Antidepressants	139 (14)	249 (12)	149 (11)
Benzodiazepines	46 (5)	87 (4)	68 (3)
Major tranquilisers	26 (3)	65 (3)	46 (3)
Other	338 (35)	741 (37)	440 (32)
Multiple drug groups	143 (15)	332 (16)	226 (16)
Received psychosocial assessment***	*n* = 1664	*n* = 4078	*n* = 2208
No	443 (27)	2026 (50)	731 (33)
Yes	1221 (73)	2052 (50)	1475 (67)
Missing	0 (0)	0 (0)	2 (0)
Admitted to hospital***	*n* = 1664	*n* = 4078	*n* = 2208
No	360 (22)	2352 (58)	921 (42)
Yes	1300 (78)	1489 (37)	1211 (55)
Missing	4 (0)	237 (6)	76 (3)
Panel C: Self-harm rate per 1000 population			
Age			
10–18	4.97	7.98	7.00
19–29	6.27	7.02	10.70
30–39	3.81	6.82	6.89
40–49	4.25	9.15	7.26
50–59	2.22	5.04	4.02
60–69	0.98	1.28	1.87
70+	0.61	0.88	0.50
Total	3.61	6.29	5.98

*p-value < 0.05; **p-value < 0.01; ***p-value < 0.0001; # other methods include: drowning, gunshot, gas, head banging.

As Table 2 shows, there were an estimated 228 075 self-harm presentations (39% males and 61% females) by 159 857 patients in 2013 in England. The highest proportion of self-harm presentations among males was in the 40–49 year age group (30%), while for females the 19–29 year age group had the highest percentage of presentations (28%). Based on the two univariate sensitivity analyses, estimated self-harm presentations in England were 215 588 after adjusting for suicide rates and 225 172 after adjusting for rurality.

**Table 2.** Estimated incidence of self-harm in England in 2013 by gender and age group

Age	10–18	19–29	30–39	40–49	50–59	60–69	70+	Total
Episodes								
Males	8911 (10%)	19 950 (23%)	16 782 (19%)	26 218 (30%)	10 654 (12%)	3878 (4%)	1644 (2%)	88 038 (100%)
Females	30 040 (21%)	38 805 (28%)	24 460 (17%)	26 904 (19%)	13 951 (10%)	3602 (3%)	2274 (2%)	140 037 (100%)
Total	38 951 (17%)	58 756 (26%)	41 242 (18%)	53 123 (23%)	24 605 (11%)	7480 (3%)	3918 (2%)	228 075 (100%)
Patients								
Males	7487 (12%)	15 629 (25%)	12 491 (20%)	14 978 (24%)	7663 (12%)	3180 (5%)	1586 (3%)	63 014 (100%)
Females	22 418 (23%)	23 929 (25%)	16 210 (17%)	18 553 (19%)	10 343 (11%)	3291 (3%)	2099 (2%)	96 843 (100%)
Total	29 905 (19%)	39 559 (25%)	28 701 (18%)	33 531 (21%)	18 006 (11%)	6470 (4%)	3685 (2%)	159 857 (100%)
Episodes (sensitivity analysis-suicide rate adjustment)								
Males	9233 (10%)	20 670 (23%)	17 387 (19%)	27 164 (30%)	11 038 (12%)	4018 (4%)	1703 (2%)	91 213 (100%)
Females	26 681 (21%)	34 465 (28%)	21 724 (17%)	23 895 (19%)	12 391 (10%)	3199 (3%)	2020 (2%)	124 375 (100%)
Total	35 913 (17%)	55 135 (26%)	39 111 (18%)	51 059 (24%)	23 429 (11%)	7217 (3%)	3723 (2%)	215 588 (100%)
Episodes (sensitivity analysis-rural area adjustment)								
Males	8749 (10%)	19 817 (23%)	16 685 (19%)	25 755 (30%)	10 415 (12%)	3762 (4%)	1592 (2%)	86 775 (100%)
Females	29 575 (21%)	38 633 (28%)	24 302 (17%)	26 474 (19%)	13 687 (10%)	3511 (3%)	2218 (2%)	138 397 (100%)
Total	38 324 (17%)	58 450 (26%)	40 987 (18%)	52 229 (23%)	24 099 (11%)	7273 (3%)	3810 (2%)	225 172 (100%)

The estimated hospital cost of self-harm in England in 2013 was approximately £128.6 (95% CI 117.8−140.9) million. In absolute terms, the majority of costs were for episodes involving women and were greatest in the Midlands and East regions (Table 3). The total hospital costs of self-harm reduced to £121.6 (95% CI 111.6−133.4) million or £127 (95% CI 116.4−139.7) million after independently adjusting for suicide rates and rurality, respectively, and assuming that the representativeness of the patients recorded in the Multicentre Study of Self-harm to all patients who self-harmed in England in the same period was not perfect.

**Table 3.** Hospital cost of self-harm across large geographic areas in England (£, 2013)

	Males	Females	Total
	Mean (95% CI)	Mean (95% CI)	Mean (95% CI)
Main analysis			
England	49 559 150 (43 896 127 to 56 429 207)	79 046 705 (70 310 153 to 89 561 701)	128 605 855 (117 835 026 to 140 934 979)
North of England	13 887 113 (12 303 350 to 15 805 226)	22 301 908 (19 843 575 to 25 260 926)	36 189 021 (33 162 982 to 39 653 206)
Midlands and East of England	14 893 788 (13 193 223 to 16 957 069)	23 776 299 (21 161 598 to 26 925 731)	38 670 087 (35 442 360 to 42 360 185)
London	8 189 719 (7 251 950 to 9 326 903)	12 933 335 (11 470 902 to 14 688 055)	21 123 054 (19 331 828 to 23 179 317)
South of England	12 588 530 (11 149 072 to 14 336 034)	20 035 163 (17 831 861 to 22 684 533)	32 623 693 (29 894 968 to 35 745 008)
Sensitivity analysis-suicide rate adjustment			
England	51 388 469 (45 485 739 to 58 231 335)	70 237 351 (62 504 833 to 79 697 099)	121 625 820 (111 606 263 to 133 361 503)
North of England	16 404 748 (14 524 117 to 18 586 623)	20 697 182 (18 424 508 to 23 477 638)	37 101 930 (34 065 729 to 40 658 786)
Midlands and East of England	15 052 615 (13 327 277 to 17 055 894)	19 248 590 (17 140 557 to 21 828 818)	34 301 205 (31 492 938 to 37 593 516)
London	8 196 243 (7 256 170 to 9 285 758)	12 939 213 (11 486 205 to 14 715 577)	21 135 456 (19 358 303 to 23 219 012)
South of England	13 383 772 (11 848 737 to 15 170 634)	20 571 587 (18 318 112 to 23 333 386)	33 955 359 (31 155 554 to 37 244 280)
Sensitivity analysis-rural area adjustment			
England	49 040 989 (43 497 765 to 55 798 083)	78 221 350 (69 455 479 to 88 833 331)	127 262 339 (116 429 823 to 139 715 863)
North of England	13 933 703 (12 359 729 to 15 851 916)	22 331 294 (19 836 612 to 25 351 426)	36 264 997 (33 188 797 to 39 811 304)
Midlands and East of England	14 390 659 (12 763 144 to 16 373 060)	23 087 662 (20 516 168 to 26 206 271)	37 478 321 (34 304 814 to 41 130 411)
London	8 721 310 (7 731 182 to 9 910 935)	13 595 724 (12 037 830 to 15 485 045)	22 317 035 (20 397 794 to 24 524 751)
South of England	11 995 316 (10 636 940 to 13 652 368)	19 206 671 (17 064 371 to 21 806 693)	31 201 987 (28 557 106 to 34 245 763)

**Fig. 1.**
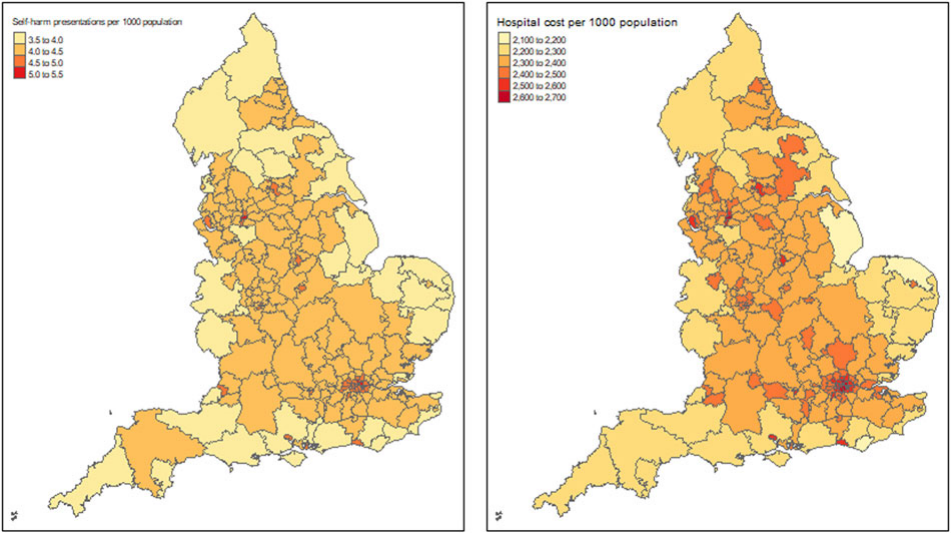
Map of England with the estimated self-harm episodes and associated hospital cost per 1000 population in 2013.

Figure 1 presents the distribution of estimated self-harm presentations and associated hospital costs per 1000 population across local health authorities in England. As shown in the figure, the incidence of self-harm and associated hospital costs was relatively lower in the majority of coastal areas, higher in inland areas and highest in the greater London area. The estimated hospital costs by CCG in England are presented in Appendix 6.

## Discussion

This study provides the first detailed estimates of self-harm presentations to hospitals and their associated hospital costs across England. The results of this study may assist national and local health decision makers in planning the distribution of funds for self-harm and prioritising interventions in areas with the highest need for tackling self-harm. Providing the incidence of self-harm presentations in each CCG by gender and age highlights sub-populations potentially where additional resources might be targeted to interventions that may prevent self-harm and assist those who have self-harmed, reducing therefore suicide deaths.

Using our incidence estimates and considering that there were 4727 (3688 male and 1039 female) deaths by suicide in England in 2013 (Statistics, 2016), our results indicate that there were 48 (24 male and 135 female) self-harm presentations to hospitals per suicide and 34 (17 male and 93 female) patients presenting with self-harm per suicide. While these ratios may seem quite large, self-harm is the strongest factor associated with subsequent suicide (Hawton *et al*., 2015). Risk is also particularly high in the period shortly after self-harm (Hawton *et al*., 2019). Therefore, primary and secondary prevention interventions that focus on reducing self-harm presentations and on provision of effective aftercare for those who do self-harm may prevent subsequent deaths by suicide (Hawton *et al*., 2013; Carroll *et al*., 2016; Geulayov *et al*., 2018). This is in line with economic evidence that supports the provision of public health interventions (including psychological therapies) for self-harm and suicide prevention (McDaid *et al*., 2017; Campion and Knapp, 2018). However, effective implementation of self-harm and suicide prevention strategies at local level is challenging in terms of both deciding what initiatives may be effective and how to evaluate these (Saunders and Smith, 2016; Hawton and Pirkis, 2017). In England, Public Health England and CCGs also have to contend with many competing health issues. Moreover, strategies need to be implemented in partnership with multiple local health service providers, as well as the local government public health services. Compliance with national guidance is another challenge for policy makers and service commissioners. Most public health and healthcare decision making in England is made at a local level, leading to substantive variation in service delivery so that many patients still do not receive psychosocial assessment when presenting at hospital for self-harm (Geulayov *et al*., 2016).

Our estimated incidence of self-harm presentations in England (i.e. 228 075) is close to previously reported more crude estimates of 200 000 episodes per year (Geulayov *et al*., 2016). This can be contrasted with much lower rates seen in Public Health England's ‘Fingertips’ database suggesting that this underestimates overall rates of self-harm by approximately 60% compared with rates based on the Multicentre Study (Clements *et al*., 2016). This is because Fingertips only includes self-harm episodes resulting in hospital admission based on Hospital Episodes Statistics data. It should be noted that our study has estimated only the incidence of self-harm presentations to hospitals; it is well recognised that much self-harm occurs in the community without presentation to hospital, especially among adolescents (Geulayov *et al*., 2018).

We estimated the hospital cost of self-harm in England in 2013 to be approximately £128.6 million (£133.8 million in 2017 prices using an inflation rate of 1.04062 based on the Hospital and Community Health Services inflation index) (Curtis and Burns, 2017). This figure is lower than the roughly estimated £161.8 million per year cost of self-harm to NHS hospitals reported recently (Tsiachristas *et al*., 2017). It also seemed robust after performing two sensitivity analyses that accounted for the association of self-harm rates with suicide rates (Geulayov *et al*., 2016) and rural areas (Harriss and Hawton, 2011). The estimated costs in the Oxford CCG area in the present study was £1 565 464 and the total hospital cost of self-harm presentations to the John Radcliffe Hospital in Oxford was actually £1 280 394. These figures therefore provide us with confidence about the internal validity of our cost estimates considering that the difference is likely to be due to the costs of self-harm presentations to the Horton General Hospital, a much smaller hospital than the John Radcliffe, which is also contracted by the Oxfordshire CCG. An additional reassurance for the robustness of our estimated incidence and costs is that the five hospitals included in the Multicentre Study cover populations with a wide range of socio-economic deprivation e.g. 5 in Manchester, 55 in Derby and 166 in Oxford (IMD score range: 1 most deprived to 209 most affluent) (Department for Communities and Local Government, 2015). This variation is reassuring considering that socio-economic deprivation is associated with self-harm and suicide (Hawton *et al*., 2001).

While detailed estimates of the costs of all cases of self-harm have been made for a single hospital (Tsiachristas *et al*., 2017), this study is to our knowledge the only detailed analysis, applying a consistent methodology to estimate national self-harm costs by documenting care trajectories and measuring actual resource utilisation for all self-harm treatment costs, broken down by age, gender and means of self-harm, across multiple general hospital sites in different areas of England. A recent evaluation of the extension of hours of a liaison psychiatry service in a hospital in the south-west of England reported mean costs per emergency department self-harm attendance, including liaison psychiatry service use and inpatient care were reduced from £784 to £700 (£777−£694 in 2013/14 prices), but unlike our analysis NHS reference costs rather than a detailed resource and costing exercise were used to estimate costs (Opmeer *et al*., 2017). No attempt was made to estimate costs at a wider geographical level.

Other UK studies have concentrated on the costs of deliberate self-poisoning alone. In 2006/07 one-year costs, not including psychosocial assessment, of 1598 deliberate self-poisonings (aged >16 years) presenting to a general hospital in Nottingham were estimated using NHS reference costs to be £1.64 million or £1026 per poisoning; the authors noted that if repeated across England costs per annum would be much higher than our estimate for all self-harm costs at approximately £170 million (£192 million at 2013/14 prices) (Prescott *et al*., 2009). UK-wide costs for emergency department presenting paracetamol poisonings following the impact of a change in national guidelines on presentations at three hospitals in Edinburgh, Newcastle and London were estimated to be £48.3 million (£49.7 at 2013/14 prices), again using English NHS tariffs rather than measuring costs (Bateman *et al*., 2014). Some much older English studies also compared the costs of treating self-poisoning, including psychosocial assessment, across multiple general hospitals over periods of up to five months in the late 1990s; they highlighted substantive variations in costs in part due to type of poisoning as well as differences in care pathways (Kapur *et al*., 1999*a*, 1999*b*, 2002), estimating England wide costs of £56 million (£90 million at 2013/14 prices) (Kapur *et al*., 2003).

Information making use of the total costs of hospital presenting self-harm to estimate national costs in other high-income countries has also been limited, although access to administrative datasets linked to health insurance records in some countries potentially would allow for more detailed estimates to be produced. Data from the 2006 US Nationwide Emergency Data Sample was used to identify presentations by individuals aged 65 years and over to emergency departments, as well as hospitalisations and hospital charges (Carter and Reymann, 2014). This resulted in an estimate of almost 22 500 presentations per annum nationwide with total charges of $354 million. Other US studies have also estimated the costs of self-harm for specific population groups or for specific types of self-harm at state or national levels make use of various administrative/billing datasets. None looked at costs for all intentional self-harm (White *et al*., 2013; Ballard *et al*., 2015; Jiang *et al*., 2017). Similarly, in Australia, cost estimates have only been made for young people, with costs between 2002 and 2012 for all children aged ⩽16 years identified through the National Hospital Morbidity Database as being hospitalised for intentional self-harm estimated to be $A 64 million (£34.5 million in 2013/14 prices). In this case neither annual costs nor detailed data for different injuries were reported (Mitchell *et al*., 2018). In Japan standard healthcare tariffs were combined with nationwide acute hospital discharge data to estimate costs of 7.7 billion Yen (£39.8 million in 2013/24 prices) for all drug-poisonings in people aged over 12 years in 2008 (Okumura *et al*., 2012). This estimate did not distinguish between intentional and unintentional poisonings, nor did it include costs for patients who were not hospitalised. An in-depth analysis of costs for all patients presenting with intentional self-harm at two hospitals in Basel, Switzerland in 2003 generated mean cost of CHF 19 165; the authors also assumed nationwide costs of CHF 191 million (£112 million in 2013/24 prices), using a national conservative estimate of 10 000 hospital presenting self-harm events per annum, but noting the very limited information on self-harm rates in the country (Czernin *et al*., 2012).

The strengths of this study include the precision of identification of self-harm presentations to general hospitals through the Multicentre Study, the use of hospital cost data for all episodes in Oxford and Derby, the advanced analytical approach to extrapolate self-harm incidence and hospital costs from the Multicentre Study to England, and the extensive sensitivity analyses to address the uncertainty in the results. The main study limitations are related to the available data and include: (a) the lack of hospital cost data in Manchester, (b) cost data being limited only to care received in general hospitals, which is only a part of the overall long-term costs of self-harm (Sinclair *et al*., 2011*b*) and (c) that estimated self-harm incidence and hospital cost may have changed since 2013 due to changes in the incidence patterns (e.g. increase in incidence among young females) and services provision (e.g. there has recently been a considerable increase in provision of hospital services for self-harm patients on a 24 h seven day a week basis in England).

Our analysis can help to identify specific population groups to support within localities and also draw more attention directly to self-harm when developing local suicide and self-harm prevention and reduction strategies. A key element of our approach has been to measure resource use and costs rather than simply use published health system charges, which usually do not reflect actual costs. This will also help in more accurate evaluation of the cost-effectiveness of any interventions that may reduce self-harm events.

There is certainly a need to build on recent albeit relatively small-sized economic evaluations of actions to increase the use of psychosocial assessments (Opmeer *et al*., 2017) to help improve referral to appropriate care pathways, as well as economic evaluations of psychological and other follow-up care (O'Connor *et al*., 2017; Haga *et al*., 2018; Park *et al*., 2018). The potential economic benefits of effective interventions may also be greater than shown in these analyses, as there will be additional costs to the health sector, local government and other public agencies which may be averted by any reduction in future risk of both non-fatal and fatal self-harm events (Hawton *et al*., 2015). Although our analysis has focused on England we believe our approach could also in principle be adapted for use in the development of self-harm prevention strategies in other country contexts, particularly those where national administrative datasets that record hospital presenting self-harm are not available.

## Data

Due to constraints on the data sharing permissions of the data in the Multicentre Study of Self-harm in England, we are not allowed to share the data for public use.

## Appendix 1

**Table:** Variation in patient characteristics and clinical care by method of self-harm in the three study sites

Variable	Oxford			Manchester			Derby		
	Self-poisoning alone	Self-injury alone	Both self-poisoning and self-injury	Self-poisoning alone	Self-injury alone	Both self-poisoning and self-injury	Self-poisoning alone	Self-injury alone	Both self-poisoning and self-injury
Age (years)	*	***	*	*	***	*	*	***	*
<18	147 (13)	33 (8)	28 (25)	259 (10)	204 (16)	33 (14)	202 (12)	89 (21)	17 (17)
18–19	64 (5)	24 (6)	7 (6)	135 (5)	79 (6)	21 (9)	107 (6)	43 (10)	7 (7)
20–29	348 (30)	167 (42)	32 (28)	750 (29)	4459 (36)	76 (32)	459 (27)	122 (28)	43 (42)
30–39	190 (16)	60 (15)	23 (20)	542 (21)	246 (36)	46 (19)	303 (18)	56 (13)	14 (14)
40–49	236 (20)	51 (13)	11 (10)	531 (21)	176 (14)	44 (18)	362 (22)	80 (18)	9 (9)
50–59	106 (9)	43 (11)	9 (8)	247 (10)	75 (6)	16 (7)	158 (9)	28 (6)	7 (7)
60–69	42 (4)	11 (3)	2 (2)	68 (3)	12 (1)	1 (0)	52 (3)	10 (2)	4 (4)
70 and older	21 (2)	6 (2)	2 (2)	37 (1)	13 (1)	2 (1)	30 (2)	5 (1)	1 (1)
Missing	1 (0)	0 (0)	0 (0)	4 (0)	2 (0)	0 (0)	0 (0)	0 (0)	0 (0)
Sex	***	***		***	***		***	***	
Male	422 (37)	126 (32)	32 (28)	1095 (43)	575 (45)	85 (36)	625 (37)	159 (37)	41 (40)
Female	733 (63)	269 (68)	82 (72)	1478 (57)	691 (55)	154 (64)	1048 (63)	274 (63)	61 (60)
Received psychosocial assessment	***	***		***	***		***	***	
No	225 (19)	197 (50)	21 (18)	1232 (48)	720 (57)	74 (31)	515 (31)	182 (42)	35 (33)
Yes	930 (81)	198 (50)	93 (82)	1341 (52)	546 (43)	165 (69)	1157 (69)	250 (58)	68 (67)
Missing	0 (0)	0 (0)	0 (0)	0 (0)	0 (0)	0 (0)	1 (0)	1 (0)	0 (0)
Admitted to hospital	***	***	***	***	***	***	***	***	***
No	140 (12)	202 (51)	18 (16)	1171 (46)	1036 (82)	145 (61)	599 (36)	280 (65)	42 (41)
Yes	1011 (88)	193 (49)	96 (84)	1262 (49)	139 (11)	88 (37)	1015 (61)	139 (32)	57 (56)
Missing	4 (0)	0 (0)	0 (0)	140 (5)	91 (7)	6 (2)	59 (3)	14 (3)	3 (3)

**p*-value < 0.05; ***p*-value < 0.01; ****p*-value < 0.0001; # other methods include: drowning, gunshot, gas, head banging.

## Appendix 2

**Table:** Variation in patient characteristics of those who received psychosocial assessment by site

Variable	Oxford	Manchester	Derby
	*n* (% of 894)	*n* (% of 1647)	*n* (% of 1087)
Age (years)***			
<18	143 (16)	128 (8)	197 (18)
18–19	65 (7)	116 (7)	70 (6)
20–29	235 (26)	556 (34)	260 (24)
30–39	141 (16)	342 (21)	193 (18)
40–49	154 (17)	294 (18)	206 (19)
50–59	92 (10)	149 (9)	95 (9)
60–69	38 (4)	36 (2)	42 (4)
70 and older	26 (2)	26 (2)	24 (2)
Missing	0 (0)	0 (0)	0 (0)
Sex***			
Male	346 (39)	727 (44)	406 (37)
Female	548 (61)	920 (56)	681 (63)
Occupational status***			
Unemployed/household	219 (24)	774 (47)	367 (34)
Employed	288 (32)	407 (25)	254 (23)
Disabled/retired	152 (17)	76 (5)	51 (5)
Student	188 (21)	268 (16)	227 (21)
Missing	47 (5)	122 (7)	188 (17)
Ethnicity***			
White	789 (88)	1398 (85)	830 (77)
Black	14 (2)	42 (3)	10 (1)
Asian	28 (3)	80 (5)	22 (2)
Other	44 (5)	97 (6)	35 (3)
Missing	19 (2)	30 (2)	190 (17)
Number of self-harm repetitions			
0	748 (84)	1460 (83)	943 (82)
1	95 (11)	186 (10)	134 (11)
2	23 (2)	51 (3)	52 (4)
>2	28 (3)	67 (4)	40 (3)
	Mean (s.d.) min−max *n*	Mean (s.d.) min−max *n*	Mean (s.d.) min−max *n*
Age	34 (16) 12–97 894	33 (14) 8–931 647	33 (16) 10–931 087
IMDS***	16 (11) 1–59 838	40 (19) 2–801 574	26 (16) 1–661 036
Number of repetitions	0.33 (1.18) 0–19 894	0.36 (1.22) 0–181 647	0.36 (1.07) 0–171 087

**p*-value < 0.05; ***p*-value < 0.01; ****p*-value < 0.0001.

## Appendix 3

**Table:** Variation in the provision of clinical care between the three study sites

Variable	Self-harm method	Self-harm method	Self-injury method	Self-injury method	Self-poisoning method	Self-poisoning method
	Model 1	Model 2	Model 3	Model 4	Model 5	Model 6
	Dependent: Assessed	Dependent: Admitted	Dependent: Assessed	Dependent: Admitted	Dependent: Assessed	Dependent: Admitted
	OR (s.e.) *p*-value [95% CI]	OR (s.e.) *p*-value [95% CI]	OR (s.e.) *p*-value [95% CI]	OR (s.e.) *p*-value [95% CI]	OR (s.e.) *p*-value [95% CI]	OR (s.e.) *p*-value [95% CI]
Site (ref: Oxford)						
Manchester	0.48 (0.05) <0.001 [0.39;0.59]	0.15 (0.02) <0.001 [0.12;0.19]	0.80 (0.15) 0.231 [0.55;1.16]	0.12 (0.03) <0.001 [0.08;0.19]	0.35 (0.05) <0.001 [0.26;0.46]	0.14 (0.02) <0.001 [0.10;0.20]
Derby	0.81 (0.08) 0.043 [0.66;0.99]	0.25 (0.03) <0.001 [0.20;0.31]	1.05 (0.20) 0.791 [0.72;1.53]	0.40 (0.08) <0.001 [0.27;0.58]	0.67 (0.09) 0.003 [0.52;0.87]	0.21 (0.03) <0.001 [0.15;0.28]
Age (years)***	1.01 (0.00) 0.005 [1.00;1.01]	1.00 (0.00) 0.559 [1.00;1.01]	1.01 (0.01) 0.040 [1.00;1.02]	1.00 (0.01) 0.366 [1.00;1.01]	1.00 (0.00) 0.156 [1.00;1.01]	1.00 (0.00) 0.700 [1.00;1.01]
Female (ref: Male)	0.96 (0.07) 0.588 [0.84;1.10]	1.25 (0.09) 0.002 [1.09;1.43]	0.65 (0.08) <0.001 [0.51;0.83]	1.42 (0.2) 0.012 [1.08;1.86]	1.10 (0.10) 0.282 [0.92;1.32]	1.19 (0.11) 0.069 [0.99;1.42]
IMDS	1.00 (0.00) 0.429 [0.99;1.00]	0.99 (0.00) <0.001 [0.99;0.99]	1.00 (0.00) 0.718 [0.99;1.01]	0.99 (0.00) 0.007 [0.98;0.99]	1.00 (0.00) 0.549 [0.99;1.00]	0.99 (0.00) <0.001 [0.98;0.99]
Number of self-harm repetitions	0.92 (0.02) 0.002 [0.87;0.97]	1.00 (0.02) 0.858 [0.95;1.04]	0.88 (0.04) 0.001 [0.81;0.95]	0.96 (0.03) 0.194 [0.91;1.02]	0.94 (0.02) 0.008 [0.90;0.98]	0.99 (0.02) 0.588 [0.95;1.03]
Admitted to hospital (ref: Not-admitted)	2.97 (0.23) <0.001 [2.56;3.45]		4.16 (0.64) <0.001 [3.07;5.63]		2.44 (0.25) <0.001 [2.00;2.98]	
Received assessment (ref: Not assessed)		2.93 (0.22) <0.001 [2.53;3.39]		3.96 (0.61) <0.001 [2.93;5.35]		2.51 (0.26) <0.001 [2.06;3.07]
Self-harm method (ref: Self-poisoning)						
Self-injury	0.79 (0.06) 0.003 [0.68;0.92]	0.13 (0.01) <0.001 [0.11;0.16]				
Both self-injury/poisoning	1.82 (0.27) <0.001 [1.36;2.42]	0.58 (0.08) <0.001 [0.44;0.77]				
Self-injury method (ref: cut/stab)						
Jump from height			1.32 (0.56) 0.517 [0.57;3.03]	1.74 (0.76) 0.206 [0.74;4.08]		
Hanging/asphyxiation			1.42 (0.28) 0.073 [0.97;2.08]	1.19 (0.24) 0.403 [0.79;1.77]		
Traffic related			1.67 (0.70) 0.218 [0.74;3.80]	2.03 (0.88) 0.102 [0.87;4.72]		
Other method			0.43 (0.07) <0.001 [0.31;0.59]	1.90 (0.33) <0.001 [1.35;2.68]		
Self-poisoning method (ref: Paracetamol)						
Paracetamol compound					1.20 (0.24) 0.377 [0.80;1.78]	0.81 (0.17) 0.311 [0.54;1.21]
Antidepressants					1.08 (0.17) 0.618 [0.79;1.47]	0.76 (0.12) 0.081 [0.56;1.03]
Benzodiazepines					0.46 (0.10) <0.001 [0.31;0.70]	0.43 (1.00) <0.001 [0.28;0.67]
Major tranquilisers					0.80 (0.19) 0.356 [0.49;1.29]	0.73 (0.20) 0.255 [0.42;1.25]
Other					0.78 (0.09) 0.035 [0.62;0.98]	0.53 (0.07) <0.001 [0.42;0.67]
Multiple drug groups					1.10 (0.16) 0.488 [0.83;1.46]	1.29 (0.19) 0.088 [0.96;1.72]
Constant	1.66 (0.24) <0.001 [1.25;2.21]	4.64 (0.70) <0.001 [3.46;6.23]	1.16 (0.29) 0.534 [0.72;1.87]	0.52 (0.13) 0.011 [0.31;0.86]	2.47 (0.50) <0.001 [1.67;3.67]	7.26 (1.61) <0.001 [4.70;11.22]
Variance	1.01 (0.20) [0.69;1.49]	1.00 (0.19) [0.69;1.45]	1.13 (0.34) [0.63;2.03]	1.20 (0.55) [0.48;2.97]	0.93 (0.31) [0.49;1.78]	1.14 (0.29) [0.69;1.88]
Sample size	7180 episodes 5241 patients	7180 episodes 5241 patients	2281 episodes 1696 patients	2281episodes 1696 patients	3947 episodes 3143 patients	3947 episodes 3143 patients

Ref: Reference category.

## Appendix 4.

Self-harm incidence per 1000 population in the three study sites

## Appendix 5

**Table:** Market force factors (2013/14)

Variable	MFF	MFF indexed to Oxford
Oxford University Hospitals NHS Trust	1. 100 325	1.00
Central Manchester University Hospitals NHS Foundation Trust	1. 056 801	0. 960 444
Derby Hospitals NHS Foundation Trust	1. 033 263	0. 939 053

## Appendix

**Appendix 6.** Hospital cost of self-harm by local authority across England in 2013

	Main male costs	Main female costs	Main total costs
	Mean	Lower 95% CI	Higher 95% CI	Mean	Lower 95% CI	Higher 95% CI	Mean	Lower 95% CI	Higher 95% CI
England	49 559 150	43 896 127	56 429 207	79 046 705	70 310 153	89 561 701	128 605 855	117 835 026	140 934 979
North of England	13 887 113	12 303 350	15 805 226	22 301 908	19 843 575	25 260 926	36 189 021	33 162 982	39 653 206
Cheshire, Warrington and Wirral	1 100 384	973 900	1 253 603	1 771 073	1 576 101	2 005 890	2 871 457	2 630 219	3 148 052
NHS Eastern Cheshire	172 581	152 590	196 709	271 350	241 508	307 445	443 932	406 756	486 552
NHS South Cheshire	161 163	142 624	183 631	257 369	229 161	291 325	418 531	383 522	458 563
NHS Vale Royal	92 285	81 705	105 095	148 347	132 079	167 953	240 632	220 491	263 736
NHS Warrington	192 429	170 325	219 251	299 601	266 535	339 364	492 030	450 763	539 098
NHS West Cheshire	203 615	180 175	232 021	332 425	295 733	376 625	536 040	490 911	587 774
NHS Wirral	278 311	246 445	316 953	461 980	411 123	523 196	740 292	678 246	811 878
Durham, Darlington and Tees	1 064 860	943 310	1 212 447	1 728 212	1 537 858	1 956 994	2 793 072	2 559 134	3 060 861
NHS Darlington	94 699	83 856	107 826	154 630	137 669	175 018	249 329	228 506	273 235
NHS Durham Dales, Easington and Sedgefield	244 908	216 826	278 943	391 122	348 163	442 899	636 030	582 815	697 091
NHS Hartlepool and Stockton-on-Tees	258 760	229 215	294 618	420 538	374 078	476 440	679 298	622 276	744 621
NHS North Durham	223 425	197 881	254 400	359 096	319 339	406 940	582 521	533 607	638 487
NHS South Tees	243 068	215 500	276 445	402 825	358 575	456 039	645 893	592 096	707 901
Greater Manchester	2 559 987	2 268 440	2 913 143	4 093 480	3 640 695	4 635 983	6 653 467	6 097 488	7 289 990
NHS Bolton	256 698	227 604	292 036	411 165	365 986	465 501	667 864	612 242	731 435
NHS Bury	167 910	148 714	191 226	273 731	243 554	309 996	441 641	404 593	484 124
NHS Central Manchester	184 782	164 203	209 545	300 498	267 199	340 432	485 280	445 037	531 395
NHS Heywood, Middleton and Rochdale	192 181	170 357	218 679	318 467	283 591	360 325	510 648	468 257	559 397
NHS North Manchester	172 856	153 331	196 410	259 338	230 113	294 635	432 194	395 981	473 473
NHS Oldham	204 914	181 685	233 123	335 378	298 741	379 294	540 292	495 539	591 474
NHS Salford	227 292	201 398	258 687	354 552	315 171	401 869	581 844	533 189	637 302
NHS South Manchester	154 637	137 174	175 698	253 576	225 015	288 044	408 213	373 682	447 831
NHS Stockport	256 317	226 817	292 054	410 542	365 203	465 090	666 859	610 738	731 161
NHS Tameside and Glossop	233 695	206 953	266 160	374 366	333 101	424 011	608 061	557 102	666 259
NHS Trafford	210 633	186 424	239 972	337 298	300 054	382 142	547 931	501 860	600 655
NHS Wigan Borough	298 072	263 767	339 649	464 570	413 193	526 343	762 642	698 556	835 723
Lancashire	1 329 281	1 177 491	1 513 586	2 132 058	1 898 172	2 413 714	3 461 339	3 172 633	3 791 458
NHS Blackburn with Darwen	135 463	120 112	154 066	219 743	195 785	248 494	355 206	325 879	388 795
NHS Blackpool	128 026	113 283	145 898	203 922	181 585	230 764	331 948	304 208	363 641
NHS Chorley and South Ribble	158 216	139 996	180 284	243 498	216 614	275 863	401 715	368 033	440 153
NHS East Lancashire	336 060	297 641	382 673	537 924	478 880	608 929	873 984	800 958	957 412
NHS Fylde and Wyre	141 753	125 420	161 519	222 895	198 337	252 619	364 648	334 091	399 624
NHS Greater Preston	189 700	168 096	215 904	299 502	266 525	339 047	489 202	448 512	535 692
NHS Lancashire North	141 690	125 646	161 147	240 169	213 926	271 626	381 859	350 183	418 428
NHS West Lancashire	98 373	87 161	111 967	164 405	146 445	185 996	262 778	240 886	288 036
Merseyside	1 088 812	964 948	1 239 277	1 790 535	1 592 166	2 028 890	2 879 347	2 638 491	3 157 255
NHS Halton	113 806	100 814	129 581	186 149	165 630	210 812	299 955	274 785	328 858
NHS Knowsley	127 721	113 235	145 280	225 917	201 005	255 926	353 638	324 008	388 121
NHS Liverpool	449 910	399 025	511 673	734 684	652 985	832 948	1 184 594	1 085 374	1 298 390
NHS South Sefton	140 808	124 711	160 311	233 182	207 385	264 216	373 990	342 579	410 254
NHS Southport and Formby	96 350	85 276	109 748	157 140	139 945	177 957	253 491	232 255	277 848
NHS St Helens	160 217	141 828	182 481	253 463	225 468	287 148	413 680	378 966	453 374
Cumbria, Northumberland, Tyne and Wear	1 751 267	1 551 092	1 994 416	2 799 883	2 490 451	3 172 076	4 551 150	4 169 294	4 988 632
NHS Cumbria	450 027	398 135	512 795	703 067	625 750	796 408	1 153 094	1 056 739	1 263 908
NHS Gateshead	182 704	161 756	208 136	290 995	258 607	329 970	473 699	433 715	519 459
NHS Newcastle North and East	142 672	126 790	161 783	230 963	205 376	261 844	373 634	342 612	409 237
NHS Newcastle West	132 614	117 620	150 801	211 904	188 481	240 073	344 518	315 853	377 426
NHS North Tyneside	182 521	161 504	207 982	294 126	261 336	333 711	476 647	436 195	523 085
NHS Northumberland	277 485	245 637	316 004	440 888	392 285	499 667	718 373	658 026	787 644
NHS South Tyneside	133 126	117 870	151 629	218 353	194 215	247 415	351 479	321 898	385 489
NHS Sunderland	250 117	221 498	284 868	409 588	364 273	464 042	659 706	604 222	723 529
North Yorkshire and Humber	1 518 410	1 344 906	1 728 981	2 382 501	2 120 376	2 698 077	3 900 911	3 574 835	4 273 933
NHS East Riding of Yorkshire	275 978	244 154	314 450	435 460	387 841	493 023	711 439	652 087	779 534
NHS Hambleton, Richmondshire and Whitby	143 665	127 341	163 487	202 201	179 996	229 052	345 865	317 182	378 567
NHS Harrogate and Rural District	143 092	126 721	162 920	222 174	197 870	251 554	365 266	334 919	400 033
NHS Hull	244 974	217 239	278 611	383 601	340 949	434 871	628 575	576 103	688 739
NHS North East Lincolnshire	143 510	127 135	163 396	230 678	205 359	261 131	374 188	342 943	409 892
NHS North Lincolnshire	152 014	134 526	173 217	238 770	212 504	270 395	390 783	358 078	428 291
NHS Scarborough and Ryedale	94 581	83 757	107 671	152 313	135 579	172 522	246 894	226 212	270 688
NHS Vale of York	320 596	283 995	364 959	517 305	460 170	586 076	837 900	767 693	918 310
South Yorkshire and Bassetlaw	1 358 698	1 203 808	1 546 792	2 171 202	1 932 085	2 458 357	3 529 900	3 235 238	3 866 250
NHS Barnsley	217 388	192 370	247 688	344 160	306 308	389 701	561 549	514 505	615 241
NHS Bassetlaw	104 426	92 459	118 927	162 850	145 060	184 258	267 275	245 101	292 731
NHS Doncaster	278 518	246 671	317 171	437 862	389 607	495 880	716 380	656 431	784 725
NHS Rotherham	234 809	207 888	267 411	375 268	334 163	424 642	610 077	559 139	668 275
NHS Sheffield	523 556	464 363	595 365	851 062	757 212	963 915	1 374 619	1 260 388	1 506 022
West Yorkshire	2 115 414	1 874 674	2 407 489	3 432 964	3 054 606	3 886 608	5 548 379	5 086 013	6 077 627
NHS Airedale, Wharfedale and Craven	137 888	122 120	156 982	224 126	199 622	253 643	362 015	331 783	396 698
NHS Bradford City	80 385	71 481	91 123	127 329	113 540	143 885	207 715	190 799	226 969
NHS Bradford Districts	298 403	264 564	339 408	501 110	446 268	566 848	799 513	733 169	876 054
NHS Calderdale	190 630	168 751	217 116	302 095	268 897	342 005	492 725	451 506	539 784
NHS Greater Huddersfield	223 233	197 776	254 084	352 997	314 075	399 801	576 230	528 117	631 291
NHS Leeds North	178 555	158 166	203 310	291 577	259 312	330 422	470 132	430 594	515 671
NHS Leeds South and East	225 209	199 648	256 194	361 167	321 097	409 362	586 377	537 429	642 572
NHS Leeds West	305 370	270 864	347 124	513 563	456 235	582 772	818 933	750 124	898 143
NHS North Kirklees	172 257	152 675	196 037	277 105	246 887	313 320	449 362	412 153	491 920
NHS Wakefield	303 484	268 559	345 786	481 894	428 741	545 812	785 377	719 481	860 634
Midlands and East of England	14 893 788	13 193 223	16 957 069	23 776 299	21 161 598	26 925 731	38 670 087	35 442 360	42 360 185
Arden, Herefordshire and Worcestershire	1 489 935	1 319 827	1 696 517	2 349 026	2 090 771	2 659 311	3 838 961	3 518 289	4 204 805
NHS Coventry and Rugby	404 080	358 338	459 590	641 551	570 792	726 610	1 045 631	958 664	1 145 219
NHS Herefordshire	165 398	146 396	188 393	256 183	228 170	289 966	421 581	386 607	461 734
NHS Redditch and Bromsgrove	162 733	144 099	185 300	256 586	228 360	290 559	419 319	384 242	459 461
NHS South Warwickshire	236 317	209 272	269 088	366 857	326 413	415 599	603 174	552 695	660 955
NHS South Worcestershire	262 876	232 728	299 424	420 221	374 159	475 666	683 097	625 935	748 492
NHS Warwickshire North	171 223	151 568	195 030	272 640	242 724	308 645	443 863	406 769	486 263
NHS Wyre Forest	87 307	77 305	99 411	134 989	120 119	152 916	222 295	203 755	243 588
Birmingham and the Black Country	2 224 063	1 972 205	2 529 454	3 681 025	3 277 631	4 165 569	5 905 087	5 415 984	6 467 345
NHS Birmingham CrossCity	651 054	577 746	739 994	1 111 544	990 100	1 257 375	1 762 598	1 616 624	1 931 103
NHS Birmingham South and Central	182 842	162 460	207 507	319 662	284 869	361 632	502 504	461 059	550 191
NHS Dudley	283 473	250 946	322 897	452 421	402 727	512 143	735 894	674 310	806 203
NHS Sandwell and West Birmingham	446 438	396 057	507 547	723 442	643 864	819 100	1 169 880	1 072 951	1 281 204
NHS Solihull	184 239	163 169	209 780	304 408	271 157	344 414	488 648	447 920	535 496
NHS Walsall	244 332	216 460	278 128	398 287	354 752	450 529	642 619	589 198	703 747
NHS Wolverhampton	231 685	205 339	263 646	371 260	330 389	420 255	602 945	552 704	660 460
Derbyshire and Nottinghamshire	1 835 022	1 625 453	2 089 118	2 917 232	2 595 875	3 303 920	4 752 254	4 355 355	5 206 655
NHS Erewash	86 958	76 936	99 112	140 001	124 520	158 618	226 959	207 837	248 830
NHS Hardwick	99 406	87 935	113 307	156 772	139 513	177 543	256 178	234 694	280 812
NHS Mansfield & Ashfield	177 237	156 891	201 892	282 524	251 348	320 026	459 761	421 196	503 898
NHS Newark & Sherwood	103 968	92 033	118 428	165 937	147 700	187 902	269 905	247 277	295 838
NHS North Derbyshire	247 234	218 803	281 626	385 079	342 791	436 010	632 313	579 632	692 846
NHS Nottingham City	303 335	269 307	344 366	495 577	440 883	561 555	798 912	732 599	874 969
NHS Nottingham North & East	133 931	118 505	152 617	214 685	190 997	243 199	348 615	319 314	382 176
NHS Nottingham West	103 122	91 274	117 483	158 980	141 312	180 232	262 102	240 032	287 321
NHS Rushcliffe	102 917	91 112	117 224	160 152	142 532	181 382	263 069	241 083	288 285
NHS Southern Derbyshire	476 914	422 422	543 050	757 527	674 257	857 829	1 234 440	1 131 455	1 352 213
East Anglia	2 219 504	1 965 570	2 527 551	3 477 435	3 094 840	3 937 845	5 696 939	5 220 733	6 241 106
NHS Cambridgeshire and Peterborough	801 904	710 307	913 134	1 248 444	1 110 422	1 414 439	2 050 348	1 878 999	2 246 112
NHS Great Yarmouth & Waveney	183 519	162 604	208 877	292 984	260 986	331 567	476 503	436 836	521 989
NHS Ipswich and East Suffolk	354 911	314 271	404 164	554 633	493 913	627 708	909 544	833 720	996 183
NHS North Norfolk	141 171	124 911	160 921	222 018	197 803	251 386	363 189	332 966	397 904
NHS Norwich	181 815	161 022	207 050	289 772	257 416	328 659	471 587	431 936	517 056
NHS South Norfolk	208 959	185 022	237 940	329 816	293 744	373 253	538 775	493 917	590 254
NHS West Norfolk	146 906	130 054	167 286	232 028	206 551	262 813	378 934	347 250	415 335
NHS West Suffolk	200 319	177 393	228 160	307 742	273 836	348 581	508 061	465 588	556 507
Essex	1 578 815	1 397 893	1 798 085	2 535 814	2 257 034	2 871 270	4 114 629	3 770 424	4 508 926
NHS Basildon and Brentwood	227 666	201 625	259 288	374 933	333 804	424 375	602 599	552 190	660 569
NHS Castle Point, Rayleigh and Rochford	152 855	135 372	174 013	243 408	216 862	275 357	396 264	363 282	434 073
NHS Mid Essex	347 187	307 296	395 532	548 006	487 563	620 727	895 193	820 081	981 123
NHS North East Essex	276 493	244 990	314 752	445 619	396 714	504 454	722 111	661 810	791 165
NHS Southend	160 127	141 703	182 478	254 057	226 099	287 680	414 183	379 419	453 833
NHS Thurrock	151 221	133 947	172 181	240 642	214 062	272 493	391 864	359 088	429 288
NHS West Essex	263 267	233 042	299 847	429 149	381 800	486 133	692 415	634 255	759 242
Hertfordshire and the South Midlands	2 500 191	2 213 730	2 846 869	3 995 713	3 554 400	4 527 149	6 495 903	5 951 194	7 118 271
NHS Bedfordshire	393 422	348 417	448 032	622 182	553 398	704 958	1 015 604	930 396	1 113 103
NHS Corby	59 253	52 435	67 525	96 412	85 769	109 200	155 665	142 600	170 602
NHS East and North Hertfordshire	503 813	446 045	573 763	816 786	726 616	925 404	1 320 599	1 209 726	1 447 561
NHS Herts Valleys	527 262	466 807	600 607	850 272	756 251	963 342	1 377 534	1 261 655	1 510 014
NHS Luton	195 984	173 796	222 848	312 750	278 377	354 024	508 734	466 576	557 055
NHS Milton Keynes	244 520	216 508	278 505	387 091	344 082	438 797	631 610	578 568	692 261
NHS Nene	575 936	509 846	656 078	910 220	809 914	1 030 835	1 486 157	1 361 511	1 628 381
Leicestershire and Lincolnshire	1 585 588	1 404 791	1 805 136	2 562 022	2 280 612	2 900 942	4 147 610	3 801 209	4 543 664
NHS East Leicestershire and Rutland	291 755	258 306	332 289	463 068	412 466	523 957	754 823	691 914	826 810
NHS Leicester City	311 154	276 222	353 455	521 697	464 087	591 221	832 851	763 545	912 544
NHS Lincolnshire East	193 449	171 224	220 364	310 939	276 875	352 113	504 387	462 190	552 840
NHS Lincolnshire West	206 466	182 889	235 061	342 728	304 994	388 003	549 194	503 347	602 002
NHS South Lincolnshire	124 787	110 432	142 157	202 174	179 963	228 909	326 962	299 549	358 507
NHS South West Lincolnshire	108 196	95 757	123 248	175 734	156 502	198 900	283 931	260 179	311 239
NHS West Leicestershire	349 781	309 847	398 278	545 681	485 573	617 895	895 462	820 684	980 694
Shropshire and Staffordshire	1 460 671	1 293 388	1 663 534	2 258 032	2 010 121	2 556 347	3 718 703	3 408 414	4 072 923
NHS Cannock Chase	125 012	110 652	142 422	196 115	174 599	222 012	321 127	294 289	351 751
NHS East Staffordshire	115 163	101 892	131 247	178 517	158 925	202 090	293 680	269 128	321 661
NHS North Staffordshire	196 300	173 825	223 539	306 331	272 648	346 893	502 631	460 636	550 692
NHS Shropshire	280 082	248 022	318 938	427 111	380 527	483 206	707 193	648 694	774 120
NHS South East Staffs and Seisdon and Peninsular	206 793	183 116	235 491	316 894	282 026	358 942	523 688	480 120	573 631
NHS Stafford and Surrounds	140 425	124 284	159 993	209 912	186 787	237 751	350 337	321 122	383 651
NHS Stoke on Trent	239 651	212 324	272 858	373 679	332 302	423 258	613 331	562 053	671 780
NHS Telford & Wrekin	157 245	139 290	179 042	249 472	222 211	282 262	406 717	372 937	445 263
London	8 189 719	7 251 950	9 326 903	12 933 335	11 470 902	14 688 055	21 123 054	19 331 828	23 179 317
London	8 189 719	7 251 950	9 326 903	12 933 335	11 470 902	14 688 055	21 123 054	19 331 828	23 179 317
NHS Barking & Dagenham	173 890	154 258	197 695	297 786	264 919	337 293	471 676	432 282	517 087
NHS Barnet	341 981	303 010	389 260	555 678	493 426	630 328	897 659	821 917	984 728
NHS Camden	230 162	203 662	262 233	358 813	317 687	408 428	588 976	538 562	646 710
NHS City and Hackney	266 555	235 822	303 584	425 072	376 402	483 811	691 626	632 410	759 564
NHS Enfield	292 814	259 614	333 053	495 297	440 394	561 107	788 112	722 072	864 441
NHS Haringey	268 651	237 816	305 966	412 802	366 034	468 943	681 453	623 646	747 486
NHS Havering	214 486	190 025	244 157	360 729	320 964	408 608	575 214	526 990	630 875
NHS Islington	224 579	198 718	255 754	351 822	311 234	400 879	576 401	526 870	633 334
NHS Newham	332 204	294 544	377 499	489 373	434 831	555 082	821 578	753 195	898 832
NHS Redbridge	267 233	236 959	303 903	437 770	389 132	496 139	705 004	645 990	772 909
NHS Tower Hamlets	289 871	256 840	329 646	429 186	379 859	488 622	719 057	657 743	788 519
NHS Waltham Forest	260 817	230 918	297 068	405 311	359 669	460 126	666 127	609 818	730 619
NHS Brent	313 060	277 263	356 396	480 095	426 093	544 956	793 155	726 250	869 454
NHS Central London (Westminster)	174 925	154 589	199 571	234 303	207 062	267 021	409 228	374 011	449 398
NHS Ealing	337 899	299 234	384 736	514 574	456 471	584 157	852 473	780 348	934 530
NHS Hammersmith and Fulham	179 844	159 031	205 055	284 328	251 225	324 011	464 172	423 932	510 423
NHS Harrow	228 220	202 285	259 665	359 147	319 090	407 332	587 366	538 070	643 736
NHS Hillingdon	271 188	240 428	308 464	433 577	385 514	491 345	704 765	645 877	772 393
NHS Hounslow	258 975	229 308	294 950	390 177	345 967	443 163	649 152	594 155	711 611
NHS West London (Kensington and Chelsea, Queen's Park and Paddington)	220 849	195 106	252 046	329 807	291 522	375 857	550 656	502 850	605 176
NHS Bexley	211 470	187 396	240 654	360 589	321 003	408 203	572 059	524 334	627 341
NHS Bromley	286 908	253 876	326 894	471 262	418 810	534 356	758 170	694 010	832 030
NHS Croydon	342 400	303 309	389 722	575 146	511 199	651 823	917 546	840 445	1 006 788
NHS Greenwich	256 775	227 480	292 287	403 316	358 057	457 738	660 091	604 425	723 859
NHS Kingston	159 445	141 158	181 617	257 810	228 929	292 458	417 255	382 032	457 634
NHS Lambeth	330 260	292 166	376 244	502 233	444 079	572 467	832 493	760 872	914 475
NHS Lewisham	281 815	249 384	321 128	451 728	400 344	513 363	733 543	670 891	805 451
NHS Merton	196 759	174 007	224 337	305 596	270 687	347 524	502 355	459 338	551 500
NHS Richmond	179 182	158 254	204 506	280 691	248 935	318 936	459 873	420 434	504 991
NHS Southwark	307 075	271 729	349 839	481 747	426 266	548 729	788 823	721 106	866 484
NHS Sutton	182 502	161 527	207 912	297 606	264 579	337 379	480 108	439 537	526 584
NHS Wandsworth	306 924	271 250	350 056	499 964	441 146	570 668	806 888	736 075	888 466
South of England	12 588 530	11 149 072	14 336 034	20 035 163	17 831 861	22 684 533	32 623 693	29 894 968	35 745 008
Bath, Gloucestershire, Swindon and Wiltshire	1 360 008	1 203 931	1 549 215	2 150 670	1 914 702	2 434 443	3 510 678	3 217 431	3 845 762
NHS Bath and North East Somerset	164 642	145 972	187 301	272 004	242 247	307 740	436 647	400 513	478 202
NHS Gloucestershire	548 929	485 844	625 379	870 630	775 014	985 740	1 419 559	1 300 750	1 555 505
NHS Swindon	211 307	186 904	240 875	323 624	287 643	366 946	534 931	489 904	586 316
NHS Wiltshire	435 130	385 230	495 623	684 412	609 675	774 396	1 119 542	1 026 294	1 226 136
Bristol, North Somerset, Somerset and South Gloucestershire	1 321 077	1 170 431	1 503 635	2 096 805	1 865 374	2 375 688	3 417 882	3 132 206	3 745 003
NHS Bristol	421 650	373 871	479 597	663 885	589 406	753 729	1 085 535	994 433	1 190 032
NHS North Somerset	179 247	158 536	204 268	285 417	254 011	323 269	464 664	425 665	509 509
NHS Somerset	469 255	415 721	534 081	754 206	671 953	853 247	1 223 461	1 121 569	1 340 429
NHS South Gloucestershire	250 925	222 211	285 785	393 297	349 932	445 432	644 222	590 296	705 667
Devon, Cornwall and Isles of Scilly	1 470 888	1 302 697	1 674 709	2 370 677	2 110 650	2 684 156	3 841 566	3 520 033	4 210 404
NHS Kernow	466 117	412 720	530 731	759 014	675 959	859 112	1 225 131	1 122 724	1 342 896
NHS North, East, West Devon	771 619	683 596	878 533	1 238 119	1 102 074	1 401 742	2 009 738	1 841 884	2 202 026
NHS South Devon and Torbay	233 153	206 394	265 492	373 544	332 629	423 056	606 697	555 953	664 822
Kent and Medway	1 591 149	1 409 819	1 811 315	2 586 491	2 304 462	2 926 195	4 177 640	3 830 305	4 574 720
NHS Ashford	107 836	95 481	122 794	179 246	159 780	202 641	287 082	263 216	314 452
NHS Canterbury and Coastal	177 364	157 455	201 545	306 759	273 537	346 677	484 123	444 269	530 211
NHS Dartford, Gravesham and Swanley	231 298	204 860	263 330	371 832	330 866	421 112	603 130	552 651	660 792
NHS Medway	254 346	225 467	289 353	408 427	363 552	462 226	662 773	607 699	725 852
NHS South Kent Coast	181 252	160 530	206 371	283 859	252 958	321 039	465 111	426 536	509 315
NHS Swale	99 795	88 456	113 557	159 298	141 932	180 164	259 093	237 608	283 669
NHS Thanet	114 822	101 809	130 615	194 529	173 435	219 905	309 351	283 763	338 907
NHS West Kent	424 436	375 633	483 551	682 542	608 201	772 060	1 106 978	1 014 666	1 212 442
Surrey and Sussex	2 458 957	2 176 289	2 801 288	3 910 345	3 478 458	4 429 834	6 369 302	5 834 245	6 981 445
NHS Brighton & Hove	281 322	249 177	320 314	435 142	386 287	493 932	716 464	656 097	785 481
NHS Coastal West Sussex	405 677	358 967	462 182	653 552	581 643	740 738	1 059 229	970 117	1 161 531
NHS Crawley	103 638	91 725	118 069	160 826	142 703	182 608	264 463	242 060	290 006
NHS East Surrey	162 273	143 589	184 881	260 186	231 542	294 662	422 459	386 965	463 023
NHS Eastbourne, Hailsham and Seaford	151 984	134 592	173 030	250 615	223 223	283 676	402 599	369 000	441 303
NHS Guildford and Waverley	191 592	169 813	218 019	302 131	268 951	342 088	493 723	452 680	540 738
NHS Hastings & Rother	154 926	137 189	176 374	250 512	223 180	283 456	405 437	371 643	444 333
NHS High Weald Lewes Havens	147 565	130 527	168 136	238 262	212 312	269 596	385 827	353 598	422 781
NHS Horsham and Mid Sussex	204 605	181 100	233 058	327 619	291 826	370 788	532 224	487 776	583 181
NHS North West Surrey	313 628	277 273	357 608	486 200	431 785	551 734	799 828	731 970	877 549
NHS Surrey Downs	254 081	224 838	289 430	409 715	364 866	463 645	663 795	608 296	727 585
NHS Surrey Heath	87 667	77 529	99 936	135 585	120 544	153 728	223 252	204 471	244 782
Thames Valley	1 920 534	1 700 818	2 186 976	3 036 308	2 701 100	3 439 558	4 956 842	4 542 153	5 431 392
NHS Aylesbury Vale	184 797	163 568	210 488	290 629	258 621	329 144	475 426	435 577	520 969
NHS Bracknell and Ascot	127 869	113 255	145 576	205 782	183 246	232 856	333 651	305 853	365 418
NHS Chiltern	286 444	253 550	326 279	464 327	413 568	525 402	750 772	687 967	822 503
NHS Newbury and District	98 697	87 336	112 449	155 667	138 704	176 052	254 363	233 178	278 508
NHS North & West Reading	91 392	80 867	104 128	144 903	128 923	164 137	236 294	216 450	258 980
NHS Oxfordshire	607 912	538 562	692 075	957 552	851 612	1 085 016	1 565 464	1 434 527	1 715 043
NHS Slough	135 878	120 341	154 701	214 474	190 556	243 277	350 352	320 924	384 125
NHS South Reading	110 245	97 652	125 434	169 836	150 630	193 054	280 081	256 420	307 084
NHS Windsor, Ascot and Maidenhead	131 015	116 075	149 121	204 191	181 515	231 505	335 206	307 121	367 355
NHS Wokingham	146 287	129 462	166 658	228 946	203 660	259 386	375 233	343 741	411 312
Wessex	2 465 916	2 184 181	2 808 156	3 883 868	3 456 588	4 397 809	6 349 784	5 818 949	6 956 018
NHS Dorset	669 218	592 504	762 188	1 035 077	921 409	1 172 098	1 704 295	1 562 412	1 867 183
NHS Fareham and Gosport	177 556	157 162	202 278	280 366	249 505	317 570	457 922	419 526	501 965
NHS Isle of Wight	118 862	105 280	135 298	188 686	168 172	213 472	307 548	282 057	336 784
NHS North East Hampshire and Farnham	195 183	172 781	222 377	303 489	269 979	343 796	498 672	456 820	546 413
NHS North Hampshire	202 219	178 871	230 468	318 325	283 145	360 675	520 545	476 739	570 590
NHS Portsmouth	202 668	179 768	230 462	313 605	278 943	355 323	516 273	473 469	565 257
NHS South Eastern Hampshire	182 869	161 995	208 175	300 584	267 820	340 028	483 453	443 270	529 749
NHS Southampton	237 329	210 457	269 785	368 371	327 196	418 060	605 700	555 108	663 644
NHS West Hampshire	480 012	424 923	546 693	775 364	690 264	877 867	1 255 376	1 150 106	1 376 357
